# A Shocking Complication of a Pneumothorax: Chest Tube-Induced Arrhythmias and Review of the Literature

**DOI:** 10.1155/2014/681572

**Published:** 2014-07-24

**Authors:** Shaun Cardozo, Kevin Belgrave

**Affiliations:** Department of Internal Medicine, Division of Cardiology, Detroit Medical Center, Wayne State University, Detroit, MI 48201, USA

## Abstract

We describe a patient with a recent chest tube insertion leading to atrial fibrillation with rapid ventricular rate that led to multiple inappropriate internal cardiac defibrillator (ICD) shocks. This is the first reported case of this occurring in a patient with an ICD leading to inappropriate shocks. Our elderly patient with emphysema presented with a spontaneous pneumothorax and developed rapid atrial fibrillation following emergency tube thoracostomy. The patient had a single lead ICD and received multiple inappropriate shocks for the rapid ventricular rate in the therapy zone. Although medical treatment helped stabilize the patient, resolution of the atrial fibrillation occurred only after the chest tube was removed. In a patient with a chest tube or other intrathoracic catheters, maintaining a high index of suspicion that chest tube insertions can cause secondary life threatening cardiovascular complications needs to be considered. In such patients, removal of the device proves to be the most prudent treatment action.

## 1. Introduction

Pneumothorax is a common complication of emphysematous lung disease ensuing from rupture of pulmonary blebs, resulting in chest pain and dyspnea. The respiratory exam of patients with a pneumothorax reveals hyper resonant percussion of the lungs with decreased or absent breath sounds on the side of the pneumothorax. Pneumothorax occurring in patients with COPD has an increased morbidity and mortality because of decreased secondary reserve. Therefore, emergent chest tube placement is the treatment of choice. Although complication rates for tube thoracostomy can be high, cardiovascular complications are rare [1]. There have been a few reported cases with cardiovascular complications, including conduction blocks. We describe a patient with a pneumothorax treated with chest tube placement, followed by rapid atrial fibrillation refractory to medical treatment that only resolved after chest tube withdrawal.

## 2. Case Presentation

**Figure 1:**
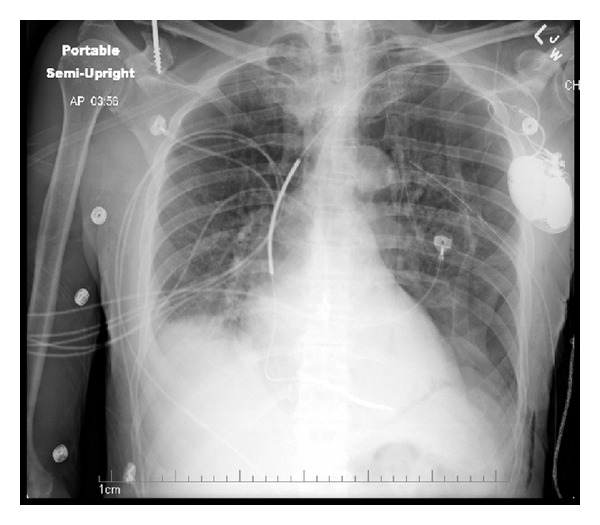
An anteroposterior view of the chest showing the chest tube in close approximation to the heart.

**Figure 2:**
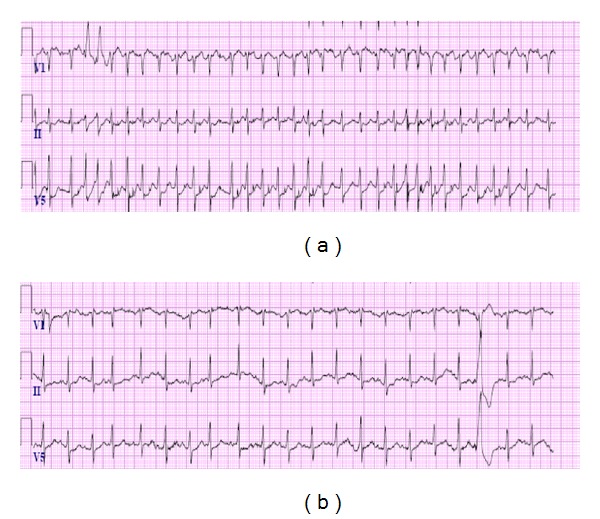
Pre- and post-ECGs after insertion and removal of chest tube and resolution of pneumothorax.

**Figure 3:**
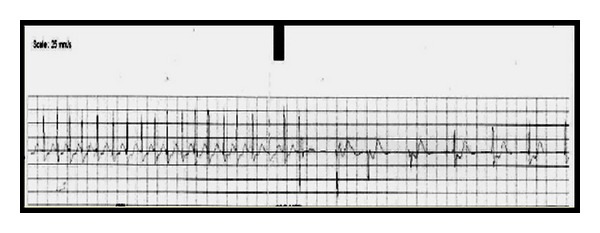
Atrial arrhythmia with ventricular rate close to 200 and 18 joule shock delivered successfully.

An 88-year-old African American gentleman with past medical history significant for emphysema, ICD placement nonischemic cardiomyopathy with an ejection fraction of 20%, presented with complaints of shortness of breath for one day. On presentation, he was already on optimal medical therapy for his heart failure, which included coreg and enalapril, and proper treatment for his COPD. He also had no prior history or documentation of significant arrhythmias. On admission, there was high suspicion for pneumothorax, which was confirmed after chest X-ray. The patient initially had an apical chest tube placed, which was found to be located within the chest wall and not working properly. Therefore, another chest tube was placed on the lateral surface of the patient's left side between the 4th and 5th intercostals spaces (Figure 1). Immediately following chest tube placement the patient developed tachycardia and was found to be in atrial fibrillation with a ventricular rate peaking around 200 (Figure 2). The patient received a total of 10 inappropriate ICD shocks over the next few hours, all of which resulted in short lived conversion followed by recurrence of the arrhythmia (Figure 3).

Intravenous amiodarone and IV esmolol drips were initiated; however, he remained in atrial fibrillation with gradual improvement of the ventricular rate to around 120–130 bpm. During his hospital stay his basic metabolic panel was stable, with his potassium ranging between 3.5 and 4.7 mMol/L. The patient's TSH was measured at 1.849 mU/L excluding other causes of an atrial arrhythmia. During hospitalization, the patient's ICD was interrogated and did not show any evidence of previous arrhythmias during the last few months and or previous ICD shocks. The patient remained hemodynamically stable throughout his hospital stay. While medical management only helped control the ventricular response, conversion to sinus rhythm only occurred after resolution of the pneumothorax and removal of the chest tube five days later.

## 3. Discussion

Spontaneous pneumothorax has the potential for many complications. Likewise, subsequent treatment with emergent chest tube placement is also associated with an overall complication rate as high as 25% [1]. The two main complications of tube thoracostomy include inability to place the tube and misplacement of the tube. Factors that predispose to complications include operator inexperience and hemodynamic instability of the patient. The other complications are related to iatrogenic complications secondary to injury of the lungs or blood vessels. These include an undrained hemothorax or pneumothorax, empyema, or local infection [2]. Cardiovascular complications, although rare, can occur. Cardiac conduction abnormalities are extremely rare following tube thoracostomy and can vary from bradyarrhythmias to tachyarrhythmias.

Our patient did have predisposing risk factors for arrhythmias which included a dilated left atrium, hypertension, and prior beta agonist use secondary to his emphysema. However, given the lack of past arrhythmias noted on device interrogation, a prior undiagnosed rhythm disorder seemed unlikely. In our patient, the arrhythmia likely resulted from mechanical trauma or stimulation from chest tube insertion, from pleuropericardial irritation, or plausibly from the pneumothorax itself. However, there are no reported cases of cardiac arrhythmias induced by a traumatic or spontaneous pneumothorax. There are studies and cases which cite ST segment changes associated with a spontaneous pneumothorax but cardiac arrhythmias [3]. Generally, these ST segment changes resolve after chest tube insertion and pneumothorax improvement. The literature supports the fact that, in all cases of spontaneous pneumothorax, the common rhythm finding is sinus tachycardia unless a massive event results in a pulseless electrical activity [4]. We do not believe that the pneumothorax caused the arrhythmia that led to the multiple ICD shocks, because the arrhythmia should have ceased after chest tube placement and management of the pneumothorax.

It is our opinion that irritation of the pericardium, by one of the former two mechanisms outlined, caused the tachyarrhythmias, which subsequently led to the patient receiving ICD shocks. Observations that support one of these mechanisms as the underlying precipitant include initiation of the arrhythmia after chest tube insertion, its resolution after chest tube removal, and the remarkable recalcitrance of the arrhythmia to repeated defibrillations while the chest tube was in place. While it cannot be ruled out entirely, chest X-ray evidence seems to rule out direct irritation of pericardium by the chest tube. Medical treatment served only to control the ventricular response and prevent further unnecessary shocks. There are now a total of six cases of chest tube-induced arrhythmias in the literature. Two cases involved bradycardia arrhythmias and three involved atrial tachyarrhythmias [1, 5, 6] (Table 1). One resulted in death from severe vagal nerve stimulation causing bradycardia and death [1]. The last reported case involved a chest tube insertion for a pneumothorax caused by a device procedure which resulted in a run of stable ventricular tachycardia [7]. The possible fatal arrhythmia only resolved after chest tube removal.

## 4. Conclusions

In conclusion, arrhythmias encountered after chest tube placement should raise the suspicion of mechanical irritation as the proximate cause. The refractory nature of the arrhythmia to medical management helps to support this suspicion. Our provocative case differs from prior reports in the fact that the arrhythmia led to another complication, multiple inappropriate shocks from a patient's ICD. The fact that there is a lack of evidence that the chest tube was directly irritating the pericardium is also interesting. This raises the concern of the interplay of a chest tube inserted into a patient with an ICD. This report emphasizes the need for cardiologists and surgeons to be watchful of potential mechanical complications resulting from chest tube placement, especially in a patient who has foreign objects, mechanical devices, or other things that could potentially aggravate cardiac problems. Maintaining a high index of suspicion that the chest tube is a possible cause of cardiac problems may prevent patients from unnecessary cardiovascular complications, some of which could be fatal.

## Figures and Tables

**Table 1:** 

Case	Age	Gender	Comorbidities	EKG	Result
1	36	Female	None	Sinus bradycardia	Death
2	54	Male	30-pack-year smoking history	High grade AV block	Resolved after chest tube was removed
3	35	Male	None, status posttrauma	Atrial fibrillation	Resolved after chest tube was removed
4	54	Male	Cirrhosis, liver transplant	Atrial fibrillation	Resolved after chest tube was removed
5	88	Male	Emphysema, heart disease, ICD	Atrial fibrillation	Resolved after chest tube was removed

6	87	Male	None	Ventricular tachycardia	Resolved after chest tube was removed
